# Integrated Transcriptomic and Metabolomic Profiling Reveals Candidate Genes and Metabolites Associated with Abdominal Fat Deposition in Pekin Ducks

**DOI:** 10.3390/ani16162491

**Published:** 2026-08-11

**Authors:** Chunyan Yang, Anqi Chen, Shuya Yang, Hao Bai, Yong Jiang, Guobin Chang, Guohong Chen, Zhixiu Wang

**Affiliations:** 1 Key Laboratory for Animal Genetics & Molecular Breeding of Jiangsu Province, College of Animal Science and Technology, Yangzhou University, Yangzhou 225009, China; yangcy202109@163.com (C.Y.); 13336295886@163.com (A.C.); ysy19990099@163.com (S.Y.); bhowen1027@yzu.edu.cn (H.B.); jiangyong@yzu.edu.cn (Y.J.); gbchang@yzu.edu.cn (G.C.); ghchen@yzu.edu.cn (G.C.); 2 Yunnan Animal Science and Veterinary Institute, Kunming 650224, China

**Keywords:** Pekin duck, abdominal fat deposition, transcriptomics, metabolomics

## Abstract

Abdominal fat deposition is an important economic trait in meat ducks, but the molecular mechanisms underlying individual variation in abdominal fat rate remain unclear. In this study, abdominal fat traits were measured in 316 male Pekin ducks, from which six ducks with high abdominal fat rates and six ducks with low abdominal fat rates were selected for transcriptomic and metabolomic analyses of abdominal adipose tissue. Considerable individual variation in abdominal fat rate was observed in the population, providing a phenotypic basis for divergent group selection. Transcriptomic analysis identified 549 differentially expressed genes, including 181 upregulated and 368 downregulated genes in the LF group relative to the HF group. Functional enrichment analysis revealed that these genes were mainly involved in lipid metabolism, fatty acid metabolism, glycerolipid and glycerophospholipid metabolism, triglyceride metabolism, cholesterol metabolism, lipid transport, and the PI3K-Akt signaling pathway. Among the representative candidate genes, *FASN, AGPAT1, ELOVL4, PEMT, PLTP, LCAT, LIPA,* and *LIPG* were upregulated, whereas *FABP5* and *FABP6* were downregulated in the HF group relative to the LF group. Metabolomic analysis detected 222 differential metabolic features. The putatively annotated metabolites among these features were mainly associated with cholesterol metabolism, primary and secondary bile acid biosynthesis, bile secretion, steroid biosynthesis, alpha-linolenic acid metabolism, PPAR signaling, and adipocytokine signaling pathways. Integrated transcriptomic and metabolomic analysis further highlighted potential gene–metabolite modules related to lipid synthesis/remodeling, cholesterol–bile acid metabolism, and lipid signaling. Overall, these findings provide an exploratory molecular framework for subsequent validation of abdominal fat deposition in Pekin ducks. The proposed candidate genes and putatively annotated metabolites require validation in independent duck populations and further functional studies.

## Simple Summary

Excessive abdominal fat deposition is undesirable in meat ducks because abdominal fat is usually discarded during processing and may reduce carcass value. However, why some ducks accumulate more abdominal fat than others remains unclear. In this study, we measured abdominal fat traits in 316 Pekin ducks and selected six ducks with high abdominal fat rates and six ducks with low abdominal fat rates for transcriptomic and metabolomic analyses of abdominal fat tissue. The results showed clear differences in gene expression and metabolite profiles between the two groups. Many differentially expressed genes were related to fatty acid metabolism, lipid transport, glycerolipid and phospholipid metabolism, triglyceride metabolism, and cholesterol metabolism. KEGG enrichment analysis indicated that the differential metabolites were associated with pathways related to cholesterol metabolism, bile acid biosynthesis, steroid biosynthesis, PPAR signaling, and adipocytokine signaling. Integrated analysis further suggested that abdominal fat deposition may be associated with coordinated changes in lipid-related genes and metabolites. These findings may provide candidate targets for subsequent genetic and functional studies of abdominal fat deposition in Pekin ducks.

## 1. Introduction

Intensive meat duck production has markedly improved growth performance, feed efficiency, and carcass yield, but these gains are often accompanied by excessive lipid deposition, particularly in abdominal adipose tissue [1,2]. As abdominal fat is largely removed during processing, its over-accumulation represents inefficient nutrient partitioning, feed energy waste, reduced carcass utilization, and economic loss [3,4]. Therefore, elucidating the molecular basis of abdominal fat deposition is essential for improving meat duck production and selection strategies.

In ducks, fat deposition varies across genetic backgrounds and adipose depots, and is shaped by coordinated regulation of adipocyte development, fatty acid metabolism, triglyceride storage, and lipid mobilization [5,6]. Zhuang et al. showed that fatty-type Pekin ducks displayed greater abdominal and subcutaneous fat accumulation, adipocyte hypertrophy, and upregulated expression of lipid-droplet-related genes, including *PLIN1, PLIN2, ANGPTL4, ATGL, ABHD5, and STK17A*, compared with lean-type ducks [7]. At the genomic level, GWAS identified *INTU* and *NUP35* as candidate genes for abdominal fat traits in Pekin ducks [8]. Our previous whole-transcriptome analyses of abdominal fat tissues from ducks with high and low abdominal fat rates identified a series of mRNAs, lncRNAs, circRNAs, miRNAs, and ceRNA networks potentially involved in abdominal fat deposition [9].

Nevertheless, transcript abundance alone cannot fully explain lipid accumulation because metabolites are closer to the final phenotype and directly reflect biochemical changes in fatty acid metabolism, glycerolipid metabolism, amino acid metabolism, and energy metabolism. Integrated transcriptomic and metabolomic analyses provide a more comprehensive strategy for linking regulatory genes with downstream metabolic changes [10,11]. Such approaches have been used to investigate adipose tissue metabolism in chickens and fat deposition in waterfowl, revealing associations among gene expression, lipid-related metabolites, and adipose tissue phenotypes [12,13]. Recent lipidomic studies in poultry have further revealed substantial differences in glycerolipids, glycerophospholipids, sphingolipids, and sterol lipids among adipose depots and animals with divergent fat-deposition phenotypes [14,15]. Similar depot-specific metabolic heterogeneity has also been observed in mammalian adipose tissues, supporting the application of metabolomic and lipidomic approaches to characterize the biochemical basis of fat deposition [16]. However, integrated transcriptome-metabolome evidence for abdominal adipose tissue in ducks with divergent abdominal fat rates remains limited.

We hypothesized that ducks with a high abdominal fat rate would exhibit coordinated changes in the expression of genes and the abundances of metabolites associated with fatty acid synthesis, lipid transport, lipid remodeling, and lipid-derived signaling. To test this hypothesis, we integrated transcriptomic and metabolomic analyses of abdominal adipose tissue from ducks with high and low abdominal fat rates. The objectives were to identify key candidate genes, metabolites, and pathways associated with abdominal fat deposition and to reveal potential gene–metabolite regulatory relationships. These findings may provide new insights into the molecular and metabolic mechanisms underlying abdominal fat accumulation in ducks and support future selection strategies aimed at reducing excessive fat deposition in meat duck production.

## 2. Materials and Methods

### 2.1. Ethical Approval

All experimental procedures were approved by the China Council on Animal Care and Ministry of Science and Technology of the People’s Republic of China. All experimental ducks were managed and handled according to the guidelines established and approved by the Animal Care and Use Committee of the Yangzhou University (No. SYDW-2019015).

### 2.2. Experimental Design and Duck Husbandry

One-day-old Pekin ducks were obtained from Dongfeng Breeding Co., Ltd. (Cangzhou, Hebei Province, China) and used in this study. All ducks were reared in a brooding system combining raised wire flooring and floor pens until 42 days of age. Throughout the experiment, the feeding and management practices adhered strictly to the “NY 5263-2004 Veterinary and Epidemic Prevention Standards for Pollution-Free Meat Duck Production” [17]. All experimental ducks were provided with feed and water ad libitum under consistent environmental conditions. The diet composition and nutrient levels are provided in Table 1.

### 2.3. Sample Collection

At 42 d of age, ducks were subjected to 12 h of feed withdrawal before slaughter. The ducks were slaughtered by venous exsanguination and subsequently defeathered. Carcass traits were measured according to the Chinese agricultural industry standard NY/T 823-2020 [18], Performance Terminology and Measurements for Poultry. After removal of the esophagus, trachea, crop, intestines, spleen, pancreas, gallbladder, gizzard contents and cuticle, reproductive organs, heart, liver, gizzard, proventriculus, and abdominal fat, the remaining carcass was weighed and recorded as the eviscerated weight. Abdominal fat was removed and weighed separately before the eviscerated weight was recorded. The abdominal fat rate was calculated according to the following formula: Abdominal fat rate (%) = [abdominal fat weight/(eviscerated weight + abdominal fat weight)] × 100.

Based on abdominal fat rate, male ducks were ranked to identify individuals representing divergent abdominal fat deposition. To minimize the potential confounding effect of sex on the transcriptomic and metabolomic profiles, only male ducks were included in the omics analyses. Six male ducks from the high abdominal fat-rate extreme were selected as the high abdominal fat group (HF, *n* = 6), and six male ducks from the low abdominal fat-rate extreme were selected as the low abdominal fat group (LF, *n* = 6). This extreme-phenotype design was used to maximize the biological contrast between the two groups and facilitate the exploratory identification of candidate molecular signals. Abdominal fat tissue samples from these 12 selected male ducks were rinsed with phosphate-buffered saline (PBS), immediately frozen in liquid nitrogen, and then stored at −80 °C until transcriptomic and metabolomic analyses.

### 2.4. RNA Extraction, Library Construction, and Sequencing

Total RNA was extracted from abdominal fat tissue samples using the Column Extraction of Tissue RNA Kit (Solarbio, R1240, Beijing, China) following the manufacturer’s instructions. RNA quality was assessed by an Agilent 2100 Bioanalyzer (Agilent Technologies, Palo Alto, CA, USA), with OD260/280 and OD260/230 ratios of approximately 1.9 and 2.1, respectively. RNA integrity was further verified by 1.0% agarose gel electrophoresis. All RNA samples had RNA integrity numbers (RINs) greater than 7.0. High-quality RNA samples were used for cDNA library construction, followed by transcriptome sequencing on an Illumina sequencing platform.

### 2.5. Transcriptome Sequencing Analysis

Raw reads were filtered using fastp (v.0.18.0) to remove adapter sequences, polyA/polyG sequences, reads containing more than 5% unknown bases, and low-quality reads. Paired-end clean reads were aligned to the Anas platyrhynchos reference genome assembly ZJU1.0 (NCBI RefSeq accession: GCF_015476345.1) using HISAT2 (v. 2.1.0) [19]. The mapped reads were assembled and quantified using StringTie (v. 1.3.4), and Gene expression levels were quantified as transcripts per million (TPM) for visualization. Raw read counts were used as input for differential expression analysis using DESeq2 (v. 1.20.0) [20] based on the raw gene-count matrix. Genes with *p* < 0.05 and fold change ≥1.5 or ≤0.67 were considered significantly differentially expressed. Gene Ontology (GO) and Kyoto Encyclopedia of Genes and Genomes (KEGG) enrichment analyses were subsequently performed to investigate their biological functions and pathways, with a nominal *p* value < 0.05 used to identify potentially enriched terms and pathways.

### 2.6. Metabolite Extraction and Profiling

Approximately 100 mg of each abdominal adipose tissue sample was thawed at 4 °C and mixed with 1 mL of pre-cooled methanol/acetonitrile/water solution (2:2:1, *v*/*v*/*v*). The mixture was vortexed, subjected to low-temperature ultrasonic extraction for 30 min, incubated at −20 °C for 10 min, and centrifuged at 14,000× *g* for 20 min at 4 °C. The supernatant was collected and vacuum-dried. Before LC-MS/MS analysis, the dried extracts were reconstituted in 100 μL of acetonitrile/water (1:1, *v*/*v*), vortexed, and centrifuged at 14,000× *g* for 15 min at 4 °C. The resulting supernatant was used for instrumental analysis.

Metabolomic profiling was performed using an Agilent 1290 Infinity LC UHPLC system coupled to an AB Sciex TripleTOF 6600 mass spectrometer (AB SCIEX, Concord, ON, Canada). Samples were separated on a Waters ACQUITY UPLC BEH Amide column (1.7 μm, 2.1 mm × 100 mm) under HILIC conditions at 25 °C, with a flow rate of 0.5 mL/min and an injection volume of 2 μL. Mobile phase A was water containing 25 mM ammonium acetate and 25 mM ammonium hydroxide, and mobile phase B was acetonitrile. The gradient was 95% B for 0–0.5 min, 95–65% B for 0.5–7 min, 65–40% B for 7–8 min, 40% B for 8–9 min, 40–95% B for 9–9.1 min, and 95% B for 9.1–12 min. Data were acquired in both positive and negative electrospray ionization modes with Gas 1 of 60, Gas 2 of 60, curtain gas of 30, source temperature of 600 °C, and ion-spray voltage of ±5500 V. The TOF MS and product ion scan ranges were 60–1000 Da and 25–1000 Da, respectively. MS/MS data were acquired in information-dependent acquisition mode with high sensitivity, a declustering potential of ±60 V, and collision energy of 35 ± 15 eV.

### 2.7. Qualitative and Quantitative Analysis of Metabol

Raw data acquired in positive- and negative-ion modes were processed separately using XCMS (v. 4.4.0), CAMERA (v. 1.56.0) and metaX (v. 1.4.1) toolbox, for data conversion, peak detection, retention-time correction, peak alignment, peak-area extraction, and isotope/adduct annotation. Metabolites were annotated by matching against public databases, including MassBank, METLIN, and MoNA, together with an in-house MS/MS spectral database. Differential metabolites between comparison groups were screened based on the variable importance in projection (VIP) values obtained from multivariate OPLS-DA and the *p* values from univariate Student’s *t*-tests. Metabolites with VIP ≥ 1 and *p* < 0.05 were considered differential metabolites. The metabolite annotations were assigned to MSI level 2. Differential metabolites were mapped to KEGG pathways for annotation and enrichment analysis. Pathway enrichment was evaluated using the hypergeometric test, and pathways with a nominal *p* value < 0.05 were considered potentially enriched.

### 2.8. Integrated Transcriptomic and Metabolomic Analysis

Based on the GO and KEGG enrichment results described in Section 2.5, pathways related to potentially associated with abdominal fat deposition were selected. All genes showing nominal differential expression and assigned to these pathways were included in the initial candidate-gene set. These pathway-related genes were subsequently imported into the STRING database for protein–protein interaction (PPI) analysis using a minimum required interaction score of 0.4. Genes forming at least one interaction within the resulting PPI network were prioritized as the final candidate genes. Genes forming interactions in the PPI network were prioritized as candidate genes, and the network was divided into five clusters using k-means clustering. Pearson correlation analysis was subsequently conducted between the expression levels of candidate genes and the relative abundances of selected differential metabolites, and correlations with a nominal *p* value < 0.05 were retained for exploratory interpretation and visualized as a heatmap.

## 3. Results

### 3.1. Phenotypic Variation in Abdominal Fat Deposition

To evaluate the variation in abdominal fat deposition within the experimental population and to provide a phenotypic basis for selecting ducks with divergent abdominal fat rates, live body weight, abdominal fat weight, and abdominal fat rate were measured in 316 Pekin ducks at 42 d of age, including 171 males and 145 females. Descriptive statistics, including the mean, standard deviation, minimum, and maximum values, were summarized separately by sex. As shown in Table 2, substantial individual variation was observed in body weight, abdominal fat weight, and abdominal fat rate. The abdominal fat rate ranged from 0.72% to 2.92% in males and from 0.72% to 3.41% in females. These descriptive results demonstrate considerable variation in abdominal fat deposition within the experimental population and provided the phenotypic basis for selecting individuals with divergent abdominal fat rates.

### 3.2. Differential Gene Expression in Abdominal Fat Tissues

**Figure 1:**
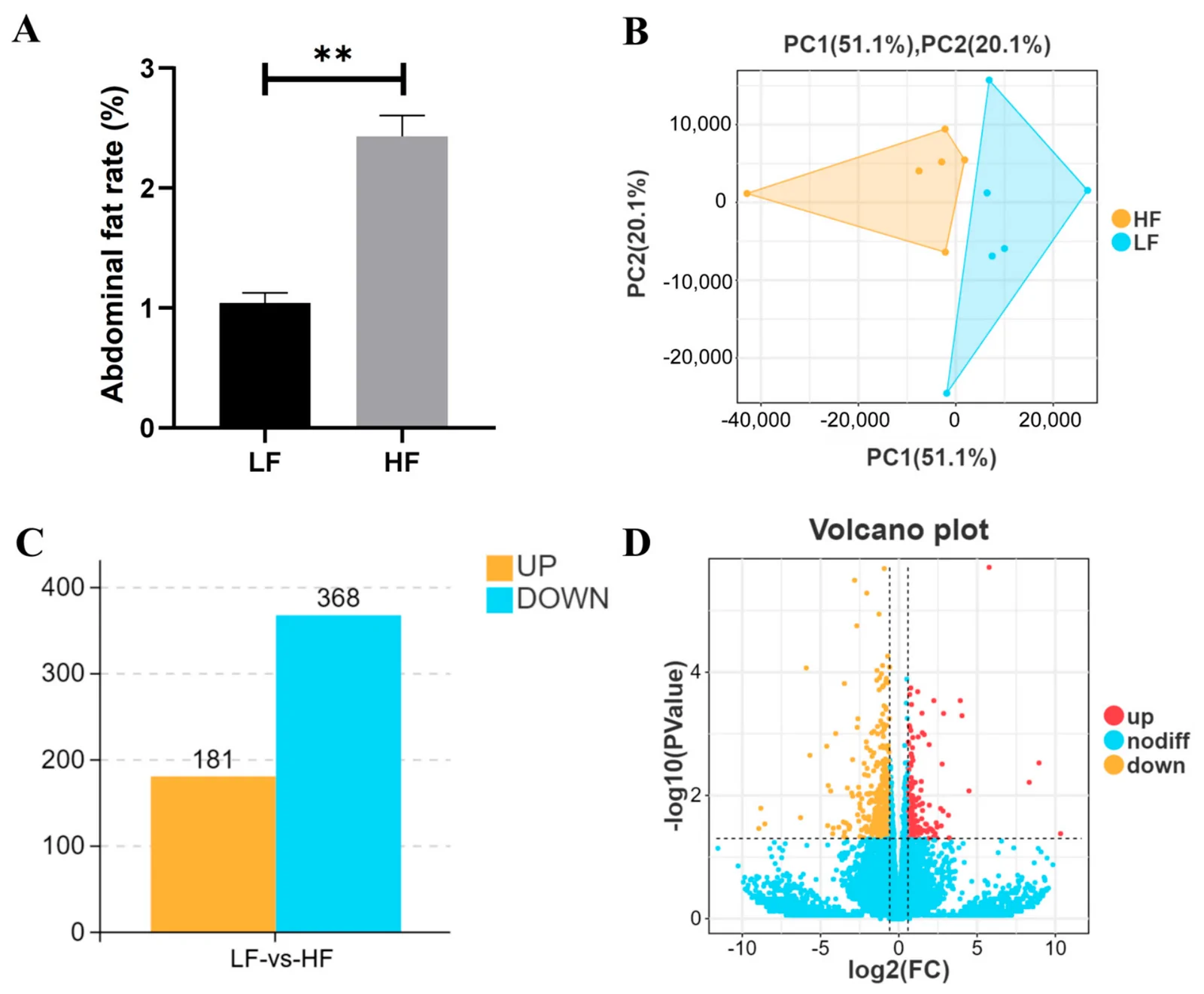
Phenotypic divergence and transcriptomic differences between LF and HF abdominal fat tissues. (**A**) Abdominal fat rate in LF and HF ducks selected for sequencing. (**B**) Principal component analysis of abdominal fat transcriptomes. ** *p* < 0.01. (**C**) Number of upregulated and downregulated DEGs. (**D**) Volcano plot of differentially expressed genes.

The abdominal fat rate of ducks selected for sequencing was significantly higher in the HF group than in the LF group (*p* < 0.01; Figure 1A), confirming clear phenotypic divergence between the two groups. Transcriptomic sequencing was performed on abdominal adipose tissue samples from six HF and six LF ducks. Each sample generated 5.57–6.92 Gb of clean data, with Q30 values ranging from 92.15% to 93.26%. The total and uniquely mapped read rates ranged from 87.10% to 89.54% and from 70.26% to 78.44%, respectively (Supplementary Tables S1 and S2), indicating that the sequencing data were of sufficient quality for downstream analyses. PCA based on transcriptomic profiles showed a visual tendency toward separation between the HF and LF groups, with PC1 and PC2 explaining 51.1% and 20.1% of the total variance, respectively (Figure 1B), indicating differences in the overall gene expression patterns of abdominal fat tissues between the two groups. Using *p* < 0.05 and fold change ≥1.5 or ≤0.67 as screening criteria, a total of 549 differentially expressed genes (DEGs) were identified in LF compared with HF, including 181 upregulated and 368 downregulated genes (Figure 1C,D).

### 3.3. Functional Enrichment and Candidate Gene Analysis of DEGs

**Figure 2:**
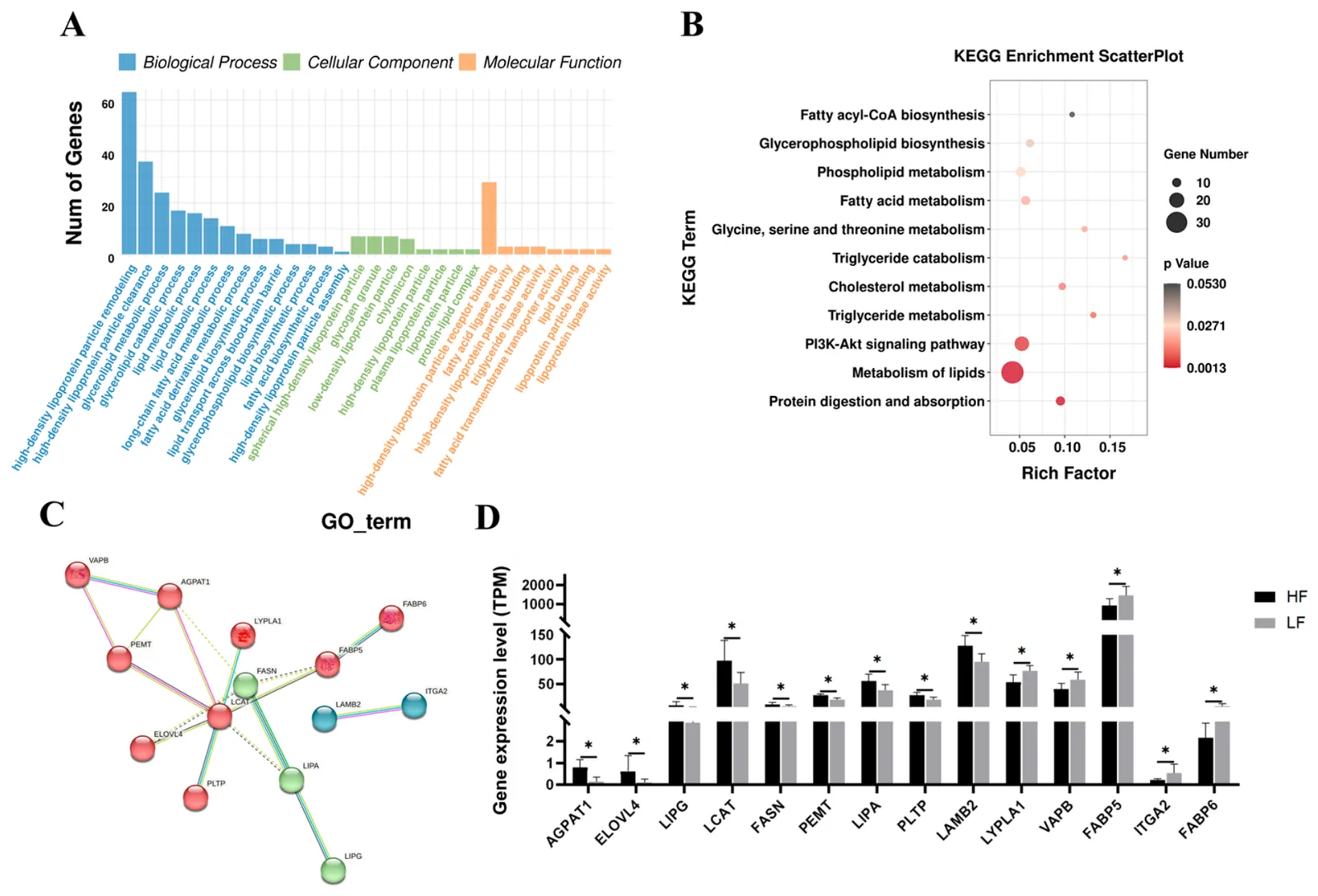
Functional enrichment and candidate gene analysis of DEGs. (**A**) GO terms enriched by DEGs between LF and HF groups. (**B**) KEGG pathways enriched by DEGs between LF and HF groups. (**C**) Protein–protein interaction network of DEGs involved in key enriched pathways. (**D**) The expression levels of representative genes are shown as TPM values. * *p* < 0.05.

To clarify the biological functions of DEGs associated with abdominal fat deposition, GO and KEGG enrichment analyses were performed. GO analysis showed that DEGs were mainly enriched in lipid-related processes, including high-density lipoprotein particle remodeling, high-density lipoprotein particle clearance, glycerolipid metabolic process, lipid metabolic process, fatty acid metabolic process, long-chain fatty acid metabolic process, and lipid transport (Figure 2A). KEGG enrichment analysis showed that DEGs were mainly involved in metabolism of lipids, fatty acid metabolism, fatty acyl-CoA biosynthesis, glycerophospholipid biosynthesis, phospholipid metabolism, triglyceride metabolism, triglyceride catabolism, cholesterol metabolism, and the PI3K-Akt signaling pathway (Figure 2B). To further identify key genes involved in these enriched pathways, pathway-related DEGs were selected for PPI network analysis. Several lipid-metabolism-related genes, including *LCAT, FASN, AGPAT1, ELOVL4, PEMT, PLTP, LIPA, LIPG, FABP5*, and *FABP6*, formed a connected interaction network (Figure 2C). The TPM expression values of these candidate genes were then extracted from the transcriptome expression matrix and visualized to compare their expression patterns between the HF and LF groups (Figure 2D). Among these candidate genes, genes related to fatty acid synthesis, phospholipid remodeling, lipid transport, and lipid hydrolysis showed distinct expression patterns between HF and LF ducks. These results suggest that abdominal fat divergence was accompanied by coordinated transcriptional changes in multiple lipid-metabolism-related processes rather than alteration of a single pathway.

### 3.4. Metabolomic Profiling and Differential Metabolite Analysis of Abdominal Fat Tissues

**Figure 3:**
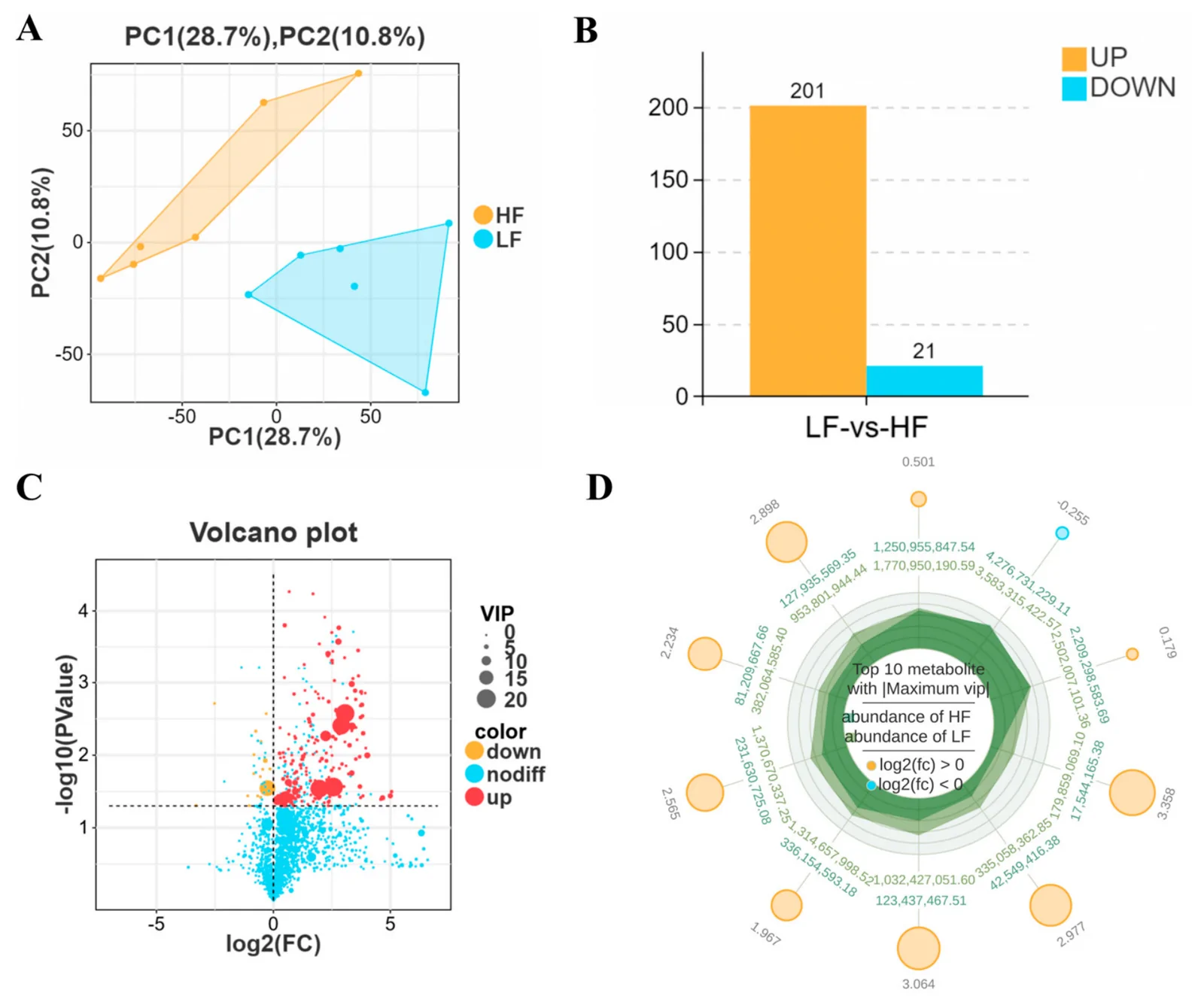
Metabolomic differences between LF and HF abdominal fat tissues. (**A**) Principal component analysis of metabolomic profiles in LF and HF groups (**B**) Number of differential metabolites between LF and HF groups. (**C**) Volcano plot of differential metabolites. (**D**) Top 10 VIP-ranked differential metabolic features between the LF and HF groups.

To determine whether divergent abdominal fat deposition was accompanied by metabolic profile differences and to identify the major metabolites contributing, metabolomic analyses were performed on abdominal fat tissues from the HF and LF groups. PCA based on metabolomic profiles showed a clear separation between the HF and LF groups, with PC1 and PC2 explaining 28.7% and 10.8% of the total variance, respectively (Figure 3A). Using VIP > 1 and *p* < 0.05 as screening criteria, a total of 222 differential metabolites were identified between the two groups, including 201 upregulated and 21 downregulated metabolites (Figure 3B). The volcano plot of differentially abundant metabolites is shown in Figure 3C. The top 10 VIP-ranked metabolites also showed distinct abundance differences between HF and LF groups, with most of them being more abundant in HF abdominal fat tissues (Figure 3D).

### 3.5. KEGG Enrichment Analysis and Abundance Patterns of Key Differential Metabolites

**Figure 4:**
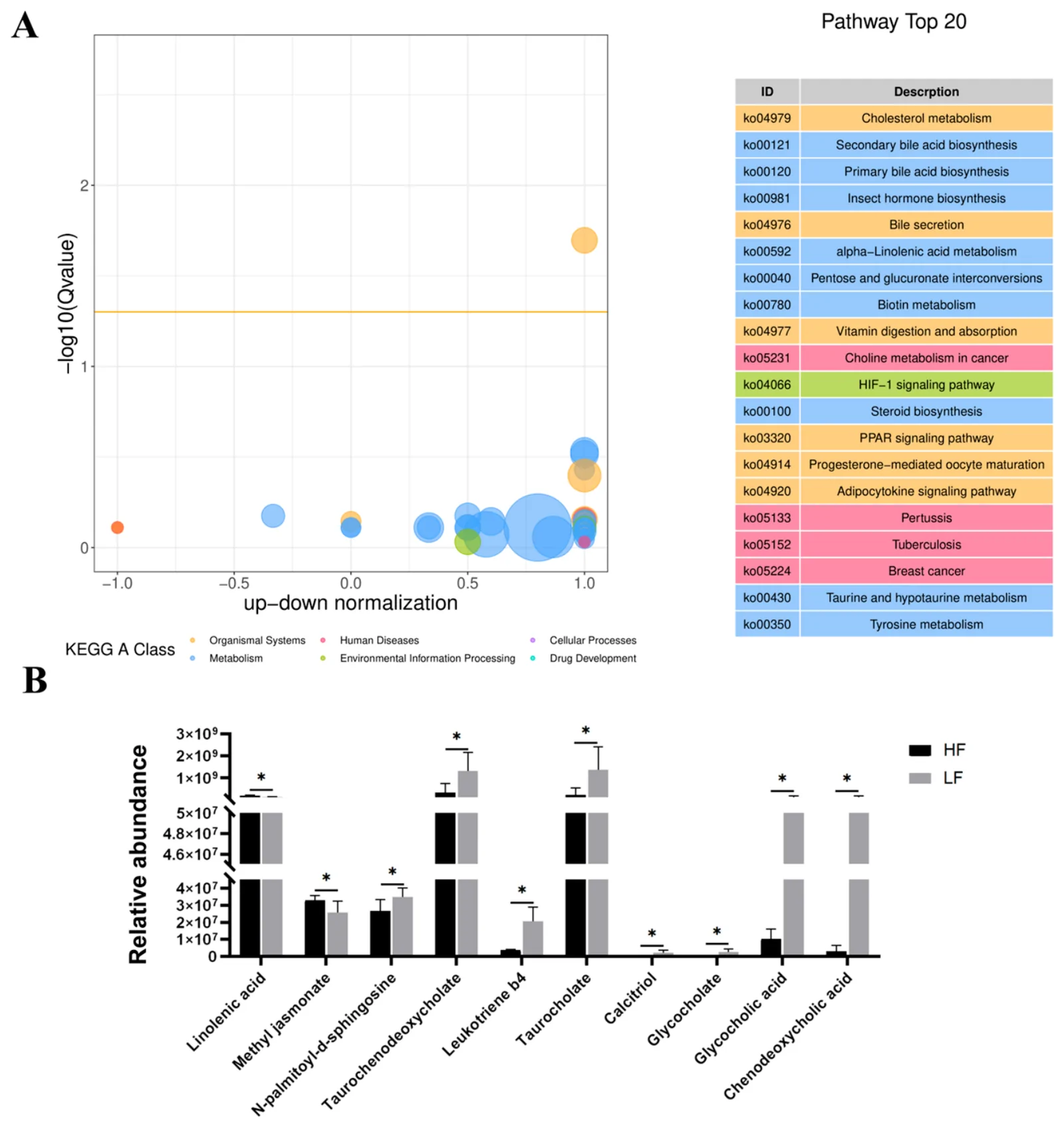
Functional enrichment and abundance patterns of key differential metabolites between LF and HF abdominal fat tissues. (**A**) KEGG pathway enrichment analysis of differential metabolites. The horizontal dashed line indicates the significance threshold of Q value = 0.05. (**B**) Relative abundance of key differential metabolites. * *p* < 0.05.

To further explore the metabolic pathways associated with abdominal fat deposition, KEGG pathway enrichment analysis was performed for the differential metabolites. The top enriched pathways were mainly related to lipid metabolism and lipid-derived signaling, including cholesterol metabolism, primary bile acid biosynthesis, secondary bile acid biosynthesis, bile secretion, steroid biosynthesis, alpha-linolenic acid metabolism, PPAR signaling pathway, and adipocytokine signaling pathway (Figure 4A). Based on these enriched pathways, several key differential metabolites were selected for further analysis, including linolenic acid, glycocholate, glycocholic acid, chenodeoxycholic acid, leukotriene B4, N-palmitoyl-D-sphingosine, taurochenodeoxycholate, calcitriol, and taurocholate. Their relative abundances were extracted from the metabolite abundance matrix and compared between the HF and LF groups (Figure 4B). Most selected bile acid-related metabolites, including glycocholate, glycocholic acid, chenodeoxycholic acid, taurochenodeoxycholate, and taurocholate, showed higher relative abundance in the HF group, suggesting that cholesterol-derived bile acid metabolism was closely associated with high abdominal fat deposition.

### 3.6. Gene–Metabolite Correlation Analysis

**Figure 5:**
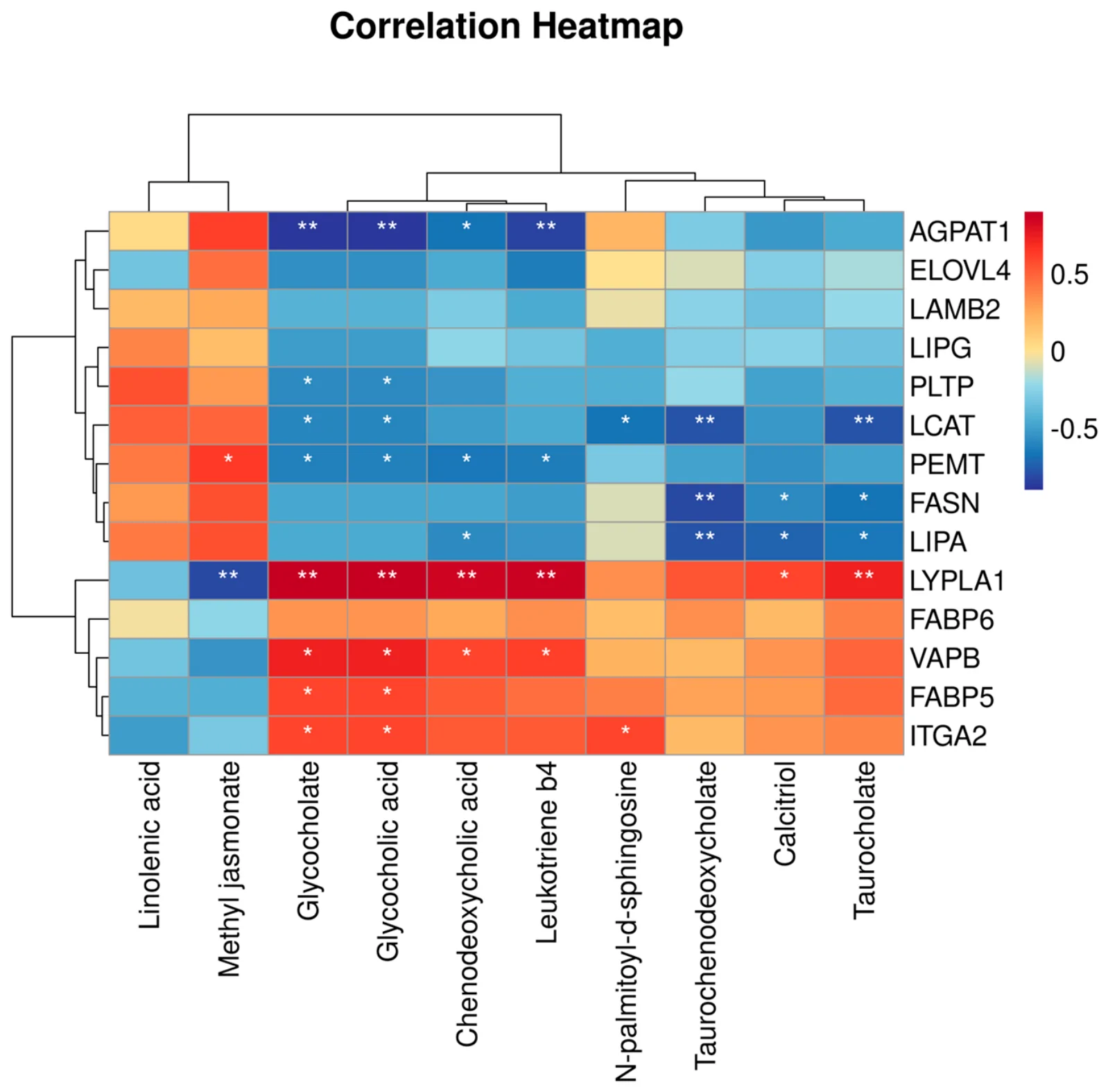
Correlation analysis between candidate genes and key differential metabolites. Note: Red and blue indicate positive and negative correlation coefficients, respectively. Rows and columns were clustered by hierarchical clustering using Euclidean distance and complete linkage. Asterisks indicate nominal, unadjusted significance levels: * *p* < 0.05 and ** *p* < 0.01.

To further investigate the relationships between transcriptomic and metabolomic changes associated with abdominal fat deposition, correlation analysis was performed between candidate genes and key differential metabolites. Correlation analysis showed that multiple candidate genes were significantly associated with key differential metabolites (Figure 5). *AGPAT1, FASN, PEMT, LIPA, PLTP*, and *LAMB2* were mainly negatively correlated with metabolites related to bile acid metabolism, cholesterol-derived metabolism, sphingolipid metabolism, and lipid signaling, including glycocholate, glycocholic acid, chenodeoxycholic acid, leukotriene B4, N-palmitoyl-D-sphingosine, taurochenodeoxycholate, calcitriol, and taurocholate, but positively correlated with linolenic acid. In contrast, *LYPLA1, FABP6, VAPB, FABP5*, and *ITGA2* showed the opposite correlation pattern. Because multiple correlations were tested without multiple-testing correction, these associations should be interpreted as exploratory and require further validation.

## 4. Discussion

Abdominal fat deposition is a complex quantitative trait. Previous studies have shown that abdominal fat pad is closely associated with total body fat content in avian species, and fat deposition in ducks is influenced by strain, sex, adipose tissue characteristics, and lipid-metabolism-related pathways. To minimize breed-related confounding effects, the present study focused on ducks with high and low abdominal fat rates within the same Pekin duck population. Considerable variation in abdominal fat rate was observed among 316 Pekin ducks reared under the same feeding and management conditions, indicating substantial individual differences in abdominal fat deposition capacity.

The transcriptomic results suggest that divergent abdominal fat deposition between HF and LF ducks was associated with coordinated changes in genes involved in lipid synthesis, storage, transport, and turnover. Rather than indicating alteration of a single pathway, the enrichment pattern pointed to changes across several interconnected stages of lipid handling, from fatty acid production and elongation to glycerolipid synthesis, membrane lipid remodeling, cholesterol transport, and lipid mobilization. These findings are consistent with previous studies showing that duck abdominal fat deposition is closely related to lipid synthesis, lipid storage, adipocyte differentiation, PPAR signaling, triglyceride metabolism, AMPK/mTOR/FoxO signaling, and energy metabolism [21,22]. Among the candidate genes, *FASN* and *ELOVL4* showed higher expression in HF ducks than in LF ducks. Because these genes participate in fatty acid synthesis and elongation, respectively, their increased expression may be consistent with greater fatty-acyl substrate availability and altered chain-length composition for lipid deposition [23,24]. *AGPAT1* and *PEMT* were also more highly expressed in HF ducks, suggesting that triglyceride synthesis, phospholipid synthesis, and membrane lipid remodeling may be associated with abdominal fat accumulation and adipocyte enlargement [25,26,27]. In parallel, the higher expression of *PLTP, LCAT, LIPA,* and *LIPG* in HF ducks may indicate changes in phospholipid transfer, lipoprotein remodeling, cholesterol esterification, lipid hydrolysis, and lipid turnover [28,29,30,31,32,33,34,35]. By contrast, *FABP5* and *FABP6* showed lower expression in HF ducks, indicating that abdominal fat deposition was not accompanied by uniform upregulation of all lipid-related genes. These divergent expression patterns may reflect altered lipid transport, lipid mobilization, feedback responses, or differences in adipocyte maturity. These expression differences suggest that the HF and LF phenotypes may reflect an altered balance between lipid synthesis and storage on the one hand and lipid transport and mobilization on the other.

The metabolomic results further showed that abdominal fat divergence between HF and LF ducks was accompanied by marked changes in lipid-related metabolic pathways. The enriched pathways collectively pointed to altered cholesterol–bile acid handling, fatty acid-derived signaling, and adipose metabolic homeostasis rather than to a change confined to a single metabolite class. In particular, the higher relative abundances of glycocholate, glycocholic acid, chenodeoxycholic acid, taurochenodeoxycholate, and taurocholate in the HF group suggest an association between high abdominal fat deposition and altered cholesterol–bile acid metabolism. In addition to serving as end products of cholesterol catabolism, bile acids can act as signaling molecules through receptors such as *FXR* and *TGR5*, thereby influencing fatty acid oxidation, glucose metabolism, energy expenditure, and adipose tissue function [36,37]. Duck bile is dominated by taurine-conjugated bile acids, and taurochenodeoxycholic acid administration in broilers increased abdominal fat deposition and altered FXR-related signaling in abdominal adipose tissue [38]. However, because the present analysis was performed in abdominal adipose tissue, the increased bile acid-related signals may reflect altered systemic transport, tissue uptake, or metabolic turnover rather than local bile acid synthesis. Changes in leukotriene B4 and N-palmitoyl-D-sphingosine further suggest that the metabolic differences between HF and LF ducks may extend beyond neutral lipid storage to inflammatory lipid signaling and sphingolipid-related metabolic states in abdominal adipose tissue [39,40]. Consistent with this interpretation, targeted lipidomic studies have revealed depot-specific differences in ceramide and sphingolipid profiles across adipose tissues [16,41], while untargeted metabolomic profiling of visceral adipose tissue has identified alterations in ceramides, sphingolipids, and glycerophospholipid-related metabolites among individuals with different metabolic phenotypes [42]. These previous findings provide additional support for the association of adipose-tissue metabolic heterogeneity with membrane-lipid remodeling and lipid-derived signaling. Moreover, the enrichment of PPAR and adipocytokine signaling pathways is consistent with differences in adipocyte differentiation, fatty acid metabolism, lipid storage, and adipose endocrine function between the two phenotypes [43]. Overall, these findings suggest that abdominal fat deposition in Pekin ducks is associated with coordinated changes in cholesterol–bile acid metabolism, fatty acid-derived signaling, sphingolipid metabolism, and adipose-related signaling pathways.

The integrated transcriptomic and metabolomic analysis further identified statistical associations between lipid-related gene-expression patterns and metabolite abundances. In the correlation network, *AGPAT1, FASN, PEMT, LIPA, PLTP*, and *LAMB2* showed similar correlation patterns with selected bile acid-, sphingolipid-, and lipid-signaling-related metabolites, whereas *LYPLA1, FABP6, VAPB, FABP5*, and *ITGA2* exhibited opposite patterns. These results revealed distinct gene–metabolite correlation patterns associated with abdominal fat deposition and suggested that variation in this phenotype may be accompanied by coordinated changes in lipid synthesis and remodeling, cholesterol–bile acid metabolism, and lipid-derived signaling.

Several limitations should be acknowledged. First, the transcriptomic and metabolomic analyses were based on six biological replicates per group, which may limit statistical power. Second, because this was an exploratory study, candidate genes were identified using a nominal *p* value < 0.05 and a fold change ≥ 1.5 or ≤0.67 without FDR correction, which may increase the risk of false-positive findings. Third, because bulk RNA-seq was performed on whole abdominal adipose tissue, the observed gene-expression differences may partly reflect differences in cellular composition rather than adipocyte-specific changes. Fourth, representative candidate genes were not validated by qPCR, and key metabolites were not independently confirmed using targeted metabolomic approaches. Moreover, correlation-based integration cannot establish causal relationships. Therefore, these findings require validation in larger independent populations and functional experiments.

## 5. Conclusions

This study identified candidate genes, metabolites, and pathways associated with divergent abdominal fat deposition in Pekin ducks. The HF and LF groups showed differences in lipid synthesis, remodeling, transport, turnover, and lipid-related metabolic signals. Integrated transcriptomic and metabolomic analyses further revealed candidate gene–metabolite associations related to abdominal fat rate. However, these findings represent exploratory associations rather than confirmed regulatory mechanisms. Independent validation and functional studies are required before these candidate molecular targets can be applied in breeding or nutritional strategies.

## Figures and Tables

**Table 1:** Ingredient composition and nutrient levels of the experimental diets.

Ingredients (%)	0–14 d	15–42 d	Nutrient Composition	0–14 d	15–42 d
Corn	46.1	42.6	Metabolizable Energy ^3^ (kcal/kg)	2793	2852
Wheat Flour	10	20.7	Crude Protein ^2^ (%)	20.14	15.58
Rice Bran	0	10	Crude fat ^2^ (%)	4.4	3.2
Soybean Meal	24	8	Calcium ^2^ (%)	0.89	0.92
DDGS	12	15	Total Phosphorus ^2^ (%)	0.62	0.66
Peanut Meal	3	0	Available Phosphorus ^3^ (%)	0.37	0.36
Oil	1	0	Lysine ^3^ (%)	1.3	0.75
Limestone Powder	1.5	1.6	Methionine ^3^ (%)	0.5	0.39
Dicalcium Phosphate	1	0.7	Threonine ^3^ (%)	0.82	0.61
Salt	0.3	0.3			
Choline chloride	0.1	0.1			
Premix ^1^	1	1			

^1^ Vitamins, minerals and nutrients supplied per kilogram of total diet are the following: Cu (CuSO_4_·5H_2_O), 10 mg; Fe (FeSO_4_·7H_2_O), 60 mg; Zn (ZnO), 60 mg; Mn (MnSO_4_·H_2_O), 80 mg; Se (NaSeO_3_), 0.3 mg; I (KI), 0.2 mg; choline chloride, 750 mg; vitamin A (retinyl acetate), 8000 IU; vitamin D3 (Cholcalciferol), 3000 IU; vitamin E (DL-α-tocopheryl acetate), 20 IU; vitamin K3 (menadione sodium bisulphate), 2 mg; thiamin (thiamin mononitrate), 1.5 mg; riboflavin, 4 mg; pyridoxine hydrochloride, 3 mg; cobalamin, 0.02 mg; calcium-D-pantothenate, 10 mg; nicotinic acid, 50 mg; folic acid, 1 mg; and biotin, 0.15 mg. ^2^ These values were determined by analysis based on triplicate determinations. ^3^ These values are as formulated.

**Table 2:** Statistics of body weight, abdominal fat weight, and abdominal fat rate in Pekin ducks.

Trait	Sex	Mean	SD	Min	Max
Body weight (g)	Male (*n* = 171)	3161.92	116.16	2770	3510
	Female (*n* = 145)	2820.49	191.58	1850	3680
Abdominal fat weight (g)	Male (*n* = 171)	38.72	9.28	20	65
	Female (*n* = 145)	36.68	10.06	13	65
Abdominal fat rate (%)	Male (*n* = 171)	1.80	0.44	0.72	2.92
	Female (*n* = 145)	1.91	0.52	0.72	3.41

## Data Availability

The raw transcriptomic sequencing data have been deposited in the NCBI Sequence Read Archive (SRA) under BioProject accession PRJNA1507848. The raw metabolomics sequence data have been deposited in the Genome Sequence Archive that are publicly accessible at https://ngdc.cncb.ac.cn/gsa, accessed on 6 August 2026 (OMIX019322).

## Supplementary Materials

The following supporting information can be downloaded at https://www.mdpi.com/article/10.3390/ani16162491/s1: Supplementary Table S1: Sequencing data quality statistics for the 12 abdominal adipose tissue RNA-seq samples; Supplementary Table S2: Read-alignment statistics for the 12 abdominal adipose tissue RNA-seq samples.
