# Supplementary material for: Integrated Transcriptomic and Metabolomic Profiling Reveals Candidate Genes and Metabolites Associated with Abdominal Fat Deposition in Pekin Ducks
Source: Animals (Basel). 2026 Aug 11;16(16):2491. doi: 10.3390/ani16162491 (PMC13508903; doi:10.3390/ani16162491)
Supplement: Supplementary file 1 [file animals-16-02491-s001.zip › Table S1 .pdf]

Table S1 Sequencing data quality statistics for the 12 abdominal adipose tissue RNA-seq samples.

| Sample | RawData(bp) | CleanData(bp) | Q20(%) | Q30(%) | N(%) | GC(%) |
|---|---|---|---|---|---|---|
| HF-1 | 6590078400 | 6321730614 | 6151812400 (97.31%) | 5864255895 (92.76%) | 159346 (0.00%) | 3343494766 (52.89%) |
| HF-2 | 6579262500 | 6283120878 | 6117265429 (97.36%) | 5831990513 (92.82%) | 159145 (0.00%) | 3264403295 (51.96%) |
| HF-3 | 6439152900 | 6161908335 | 6002487288 (97.41%) | 5728014811 (92.96%) | 112612 (0.00%) | 3289629617 (53.39%) |
| HF-4 | 6001391400 | 5766594865 | 5611899289 (97.32%) | 5347017405 (92.72%) | 144990 (0.00%) | 2948693771 (51.13%) |
| HF-5 | 6115010100 | 5923381364 | 5749840322 (97.07%) | 5458527543 (92.15%) | 146335 (0.00%) | 3010345266 (50.82%) |
| HF-6 | 7284875700 | 6921971392 | 6731942426 (97.25%) | 6408333082 (92.58%) | 127221 (0.00%) | 3575681360 (51.66%) |
| LF-1 | 6490555800 | 6233502011 | 6079193694 (97.52%) | 5803849310 (93.11%) | 157289 (0.00%) | 3179534752 (51.01%) |
| LF-2 | 7018027800 | 6725044665 | 6549207969 (97.39%) | 6249070129 (92.92%) | 169479 (0.00%) | 3545581518 (52.72%) |
| LF-3 | 7147819200 | 6880039557 | 6712641574 (97.57%) | 6411169639 (93.19%) | 122658 (0.00%) | 3533335810 (51.36%) |
| LF-4 | 6701786700 | 6387939053 | 6224560009 (97.44%) | 5945648616 (93.08%) | 175158 (0.00%) | 3255644081 (50.97%) |
| LF-5 | 5821330500 | 5572076258 | 5436307684 (97.56%) | 5196308530 (93.26%) | 140955 (0.00%) | 2781049162 (49.91%) |
| LF-6 | 6595649100 | 6309377957 | 6139651118 (97.31%) | 5851159570 (92.74%) | 158905 (0.00%) | 3127574742 (49.57%) |
