# Supplementary material for: Integrated Transcriptomic and Metabolomic Profiling Reveals Candidate Genes and Metabolites Associated with Abdominal Fat Deposition in Pekin Ducks
Source: Animals (Basel). 2026 Aug 11;16(16):2491. doi: 10.3390/ani16162491 (PMC13508903; doi:10.3390/ani16162491)
Supplement: Supplementary file 1 [file animals-16-02491-s001.zip › Table S2 .pdf]

Table S2 Read-alignment statistics for the 12 abdominal adipose tissue RNA-seq samples.

Sample	Total	Unmapped(%)	Unique_Mapped(%)	Multiple_Mapped(%)	Total_Mapped(%)
HF-1	43314896	12.90%	77.32%	9.79%	87.10%
HF-2	43084330	11.96%	75.88%	12.16%	88.04%
HF-3	41917320	12.83%	70.26%	16.91%	87.17%
HF-4	39405002	11.89%	73.72%	14.39%	88.11%
HF-5	40028248	11.69%	78.44%	9.87%	88.31%
HF-6	47512074	12.34%	74.56%	13.10%	87.66%
LF-1	42391584	11.61%	77.25%	11.14%	88.39%
LF-2	45786100	12.55%	75.10%	12.34%	87.45%
LF-3	46793616	11.55%	75.52%	12.93%	88.45%
LF-4	43864952	12.02%	76.71%	11.27%	87.98%
LF-5	37995042	10.46%	76.62%	12.92%	89.54%
LF-6	43108114	11.55%	77.22%	11.23%	88.45%
